# Demographics, Disease Characteristics, and Treatment Patterns of Patients with Plaque Psoriasis Treated with Biological Drugs: The Experience of a Single-Centre Study in Poland

**DOI:** 10.3390/jcm13247647

**Published:** 2024-12-16

**Authors:** Agnieszka Kimak-Pielas, Ewa Robak, Radosław Zajdel, Agnieszka Żebrowska

**Affiliations:** 1 Department of Dermatology and Venereology, Teaching Hospital No. 2, 90-549 Lodz, Poland; 2 Department of Dermatology, Medical University of Lodz, 90-647 Lodz, Poland; 3 Department of Economic and Medical Informatics, University of Lodz, 90-214 Lodz, Poland; 4 Department of Medical Informatics and Statistics, Medical University of Lodz, 90-645 Lodz, Poland

**Keywords:** plaque psoriasis, biological drugs, demographics, effectiveness

## Abstract

**Objectives**: This study is a retrospective analysis of patients with plaque psoriasis treated with biological drugs at a single center in Poland. We sought to evaluate patient demographics, disease characteristics, comorbidity burden, and treatment patterns in this cohort. **Methods**: Data were collected from the medical records of patients with plaque psoriasis who received biological treatments. In total, data from 1 January 2013 to 2 August 2024 were analyzed, encompassing 159 patients. The variables analyzed included age, disease duration, affected areas, prior treatments, and treatment outcomes. **Results**: The mean age at the start of biological treatment was 48 years (range: 10–73 years), with an average psoriasis duration of 18.2 years (range: 1–51 years). Obesity was noted in 39% of patients. Psoriasis lesions commonly affected the scalp (74.66%) and nails (64.38%). Methotrexate was the most commonly used systemic therapy prior to biologics (86.30%). Risankizumab and adalimumab were the most frequently prescribed biologics. Secondary treatment failure led to the highest discontinuation rates with tildrakizumab, whereas bimekizumab, guselkumab, risankizumab, and secukinumab showed the lowest rates. **Conclusions**: Biological drugs play a pivotal role in managing plaque psoriasis, particularly for patients with comorbidities and in treating challenging areas such as the scalp and nails. Risankizumab and adalimumab were prominent in prescription patterns. Future research involving larger cohorts and prospective designs is needed to deepen understanding and optimize treatment strategies for plaque psoriasis in Poland.

## 1. Introduction

Plaque psoriasis is a chronic, immune-mediated skin disease affecting up to 3% of the Polish population, with varying severity and a range of associated comorbidities, such as psoriatic arthritis and metabolic diseases [1,2]. The common underlying factor is chronic inflammation driven by the overstimulation of immune pathways, including TNF and IL-17. This contributes to insulin resistance, endothelial dysfunction, and increased cardiovascular risk [3]. The presence of comorbidities impacts not only disease progression, but may also limit treatment options, necessitating more individualized approaches.

Treatment methods for psoriasis include topicals, immunosuppressants and immunomodulators, retinoids, and, over the last two decades, biological drugs. The introduction of biologics has revolutionized the management of moderate-to-severe plaque psoriasis and has become a cornerstone in the treatment of psoriasis. Understanding the disease characteristics and treatment patterns of patients receiving biological drugs is essential for optimizing care and addressing gaps in treatment access and effectiveness.

In Poland, psoriatic patients have had access to government-funded biological therapy since 1 January 2013, under the Polish Drug Program B.47, “Treatment of moderate and severe plaque psoriasis (ICD-10 L40.0)” [4]. It is a therapeutic initiative aimed at providing access to biological treatments for patients with moderate-to-severe psoriasis who have not responded adequately to conventional therapies. Eligibility for the B.47 program is determined based on specific clinical criteria. Over the years, the program has undergone significant changes to optimize patient outcomes, including adjustments to treatment durations and broader access to newer medications, reflecting ongoing efforts to improve the standard of care for psoriasis patients in Poland. A comprehensive summary of the B.47 drug program is provided in the Supplementary Materials.

Despite advances in biological therapies, several issues remain to be clarified in the management of psoriasis with these treatments. These include identifying patient subgroups who benefit most from specific therapies, optimizing long-term treatment strategies, and addressing barriers to equitable access, particularly within healthcare systems such as Poland’s.

The objectives of this research were to provide insights into the demographics and clinical characteristics of Polish psoriasis patients receiving biological therapies, evaluate treatment outcomes, and identify areas for improvement in disease management within the Polish healthcare system. To the best of the authors’ knowledge, no in-depth studies have been conducted on real-world populations of Polish psoriasis patients treated with biological therapies.

## 2. Materials and Methods

### 2.1. Study Design

Our retrospective observational study included patients enrolled in the Polish Drug Program B.47, “Treatment of moderate and severe plaque psoriasis (ICD-10 L40.0)” between 1 January 2013 and 2 August 2024. This study included patients previously qualified for biological treatment in accordance with the requirements of the B.47 program. The criteria for psoriasis severity for enrolling in the B.47 drug program and the criteria for treatment duration have changed over time and are described in detail in the Supplementary Materials.

**Figure 1:**
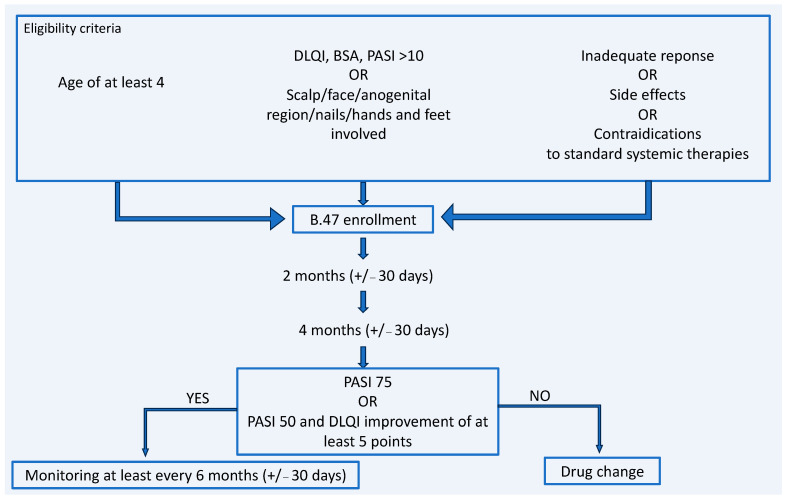
Study design.

Treatment effectiveness evaluations occurred after two months (±30 days) and four months (±30 days) following the first administration of the active substance. If therapy continued, evaluations were conducted at least once every six months (±30 days). Adequate response to treatment was defined as either a reduction in the PASI score of at least 75% or a reduction in the PASI score of at least 50% combined with an improvement in quality of life, assessed using the DLQI (or CDLQI) scale, by at least 5 points (Figure 1).

An event was classified as a primary failure if an adequate response to the administered active substance was not achieved within four months (±30 days). Secondary failure was defined as a loss of adequate response observed during two consecutive visits. The end of a treatment period was considered an administrative conclusion to therapy.

### 2.2. Patients and Baseline Information

The medical records of patients with plaque psoriasis who were enrolled in the National Health Service’s drug program B.47 between 1 January 2013 and 2 August 2024 at the dermatology department were reviewed. Patients were included in the study based on the criteria from the National Health Service’s drug program B.47. A retrospective chart review study was conducted using secondary data extracted from the patients’ medical records, including the following demographic and clinical data: age, sex, body mass index (BMI), age at onset of psoriasis, age at program enrollment, family history of psoriasis, comorbidities, psoriasis pattern, previous systemic treatments, and biological treatments within the program.

A total of 159 patients were included in the analysis. However, some patients underwent multiple cycles of treatment and are therefore represented by multiple treatment periods. Ultimately, our study group consisted of 159 patients enrolled in the drug program B.47, associated with a total of 300 drug periods.

### 2.3. Statistical Analysis

Statistical analyses were performed using Statistica 13.3 (TIBCO Software Inc., Palo Alto, CA, USA). Descriptive statistics were used to summarize patient demographics, disease characteristics, and treatment regimens. Categorical variables were presented as counts and percentages, whereas continuous variables were expressed as means with ranges or medians with IQRs, depending on the distribution of the variables (normal and non-normal, respectively). The significance level was set at *p* ≤ 0.05.

## 3. Results

As of 2 August 2024, a total of 159 patients were enrolled in the Polish Drug Program B.47, “Treatment of moderate and severe plaque psoriasis (ICD-10 L40.0)”, in our department. However, demographic, general medical, and previous treatment data were incomplete for 13 patients, leading to their exclusion from parts of the analysis. Therefore, for parts 1–3, data from 146 patients were analyzed. For Chapter 4, data from all 159 patients were included.

### 3.1. Demographics and Comorbidities at Enrollment

Among the 146 analyzed patients, there were 81 men (55.5%) and 65 women (44.5%). Two of them were children (one male and one female). All patients were Caucasian. Out of these, 66 patients (45.2%) had a family history of psoriasis. Patients enrolled in the B.47 program had a mean age of 48 years (range: 10–73 years) at the commencement of biological treatment, and their mean age of psoriasis onset was 25.6 years (range: 2–76 years). The mean duration of psoriasis prior to biological treatment in the B.47 program was 18.2 years (range: 1–51 years) (Table 1).

**Figure 2:**
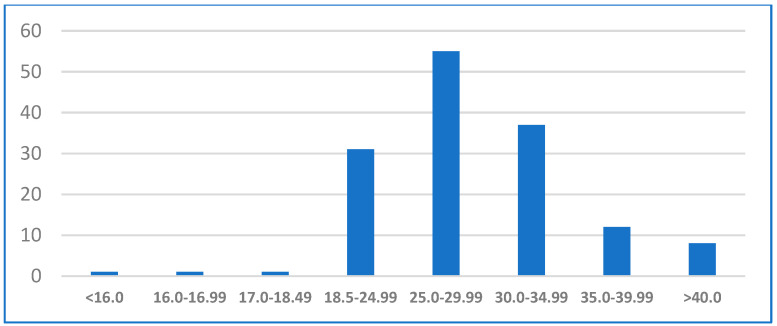
Distribution of Body Mass Index (BMI) among patients, showing the range and frequency of BMI values within the study group (*N* = 146).

The mean BMI of the patients was 28.9 (range: 15.98–44.82). Among them, 37.7% (55 patients) were overweight (BMI: 25.0–29.9) and 39.0% (57 patients) were obese (BMI: 30.0 and above) (Figure 2). The analyzed comorbidities also included arterial hypertension, type 2 diabetes mellitus, and dyslipidemia. A total of 60% of patients had at least one comorbidity, with the most common, aside from obesity, being arterial hypertension in 54 patients (37%), dyslipidemia in 40 patients (24.4%), and type 2 diabetes mellitus in 23 patients (15.8%). Twelve patients (8.22%) had all four comorbidities. Additionally, 50 patients (34.2%) reported joint pain, and 27 patients (18.5%) were current or former smokers (Table 1).

### 3.2. Baseline Psoriasis Pattern

**Figure 3:**
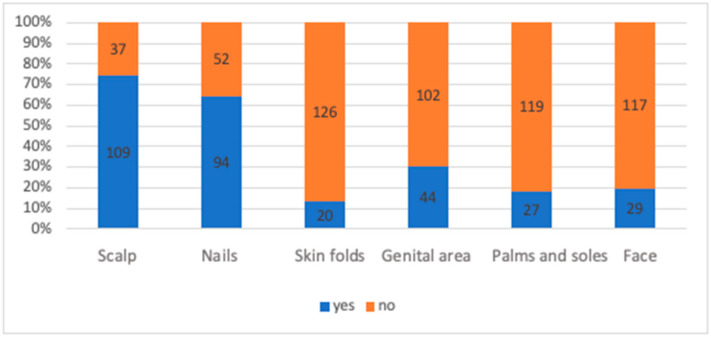
Number of patients with involvement of specific areas. Some patients had more than one area affected (*N* = 146).

Data on the exact distribution of psoriatic lesions at the time of enrollment was limited for 13 patients, leading to their exclusion from this part of the research. Consequently, 146 patients were included in this analysis. Baseline DLQI, BSA, and PASI scores were 19.38, 23.08, and 17.39, respectively (Table 2). In terms of specific areas of involvement, the most frequently affected area was the scalp (109 patients, 74.66%), followed by the nails (94 patients, 64.38%), genital area (44 patients, 30.14%), face (29 patients, 19.86%), palmoplantar region (27 patients, 18.49%), and skin folds (20 patients, 13.70%) (Figure 3). Additionally, 40 patients were enrolled in the B.47 program after meeting the criteria related to the involvement of specific areas.

### 3.3. Previous Treatment

In the analyzed group, methotrexate (MTX) was the most frequently used conventional systemic drug prior to biological treatment, administered to 126 patients (86.30%). This was followed by ciclosporin A (CsA), used by 114 patients (78.08%), and acitretin (Aci), used by 48 patients (32.88%). Phototherapy was provided to 91 patients, with 58 patients (39.73%) receiving UVB-NB (Ultraviolet B 311 nm phototherapy) and 33 patients (22.76%) receiving PUVA (Psoralen and Ultraviolet A phototherapy). Treatment success was achieved in only 13 patients on MTX, 15 patients on CsA, 1 patient on Aci, and 3 patients on PUVA. Treatment discontinuation in these cases was caused by side effects of the drugs. UVB-NB showed greater effectiveness, achieving a success rate of 34.48%. However, the improvement in skin lesions was only temporary, and continuing phototherapy sessions became difficult for some patients due to the demanding requirement of attending multiple times per week (Table 3).

Treatment with MTX, CsA, and Aci was discontinued in over half of the treated patients due to side effects. A comprehensive list of all the side effects for these drugs is presented in the Supplementary Materials. Phototherapy was generally better tolerated among patients. Side effects from PUVA therapy occurred in one-third of the patients and were associated with psoralen intake, including stomach pain, diarrhea, elevated liver enzymes, headache, and dizziness. Only three patients receiving UVB-NB (5.17%) experienced side effects, which manifested as a worsening of psoriasis symptoms.

Out of the total patients, 29 (19.86%) had previously undergone biological treatment before enrolling in the B.47 program. Of these, 23 patients had been treated with a single biological agent, 3 patients with two different biological drugs, and 3 patients with three different active substances. The biological treatments administered included etanercept, adalimumab, infliximab, ustekinumab, ixekizumab, secukinumab, tildrakizumab, risankizumab, guselkumab, and brodalumab.

### 3.4. Biological Treatment in the B.47 Program

**Figure 4:**
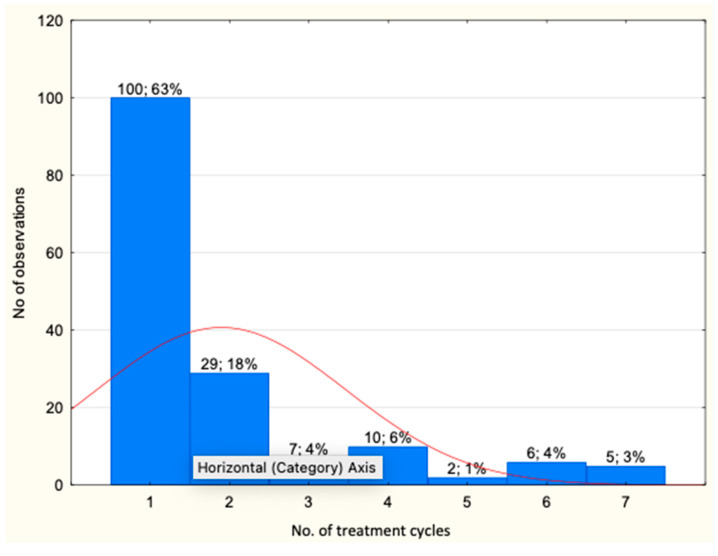
Number of drug periods in the analyzed group, the red line signifies the distribution curve (*N* = 300).

A total of 159 patients were enrolled in the program during the analyzed period. Of these, five patients had been enrolled but had not yet received their first dose of the drug (two patients were enrolled for bimekizumab and one patient each for risankizumab, secukinumab, and ixekizumab). The total exposure to biologics amounted to 300 drug periods and 131,251 patient-days. The majority of patients, 100 (63%), received one drug period, whereas 29 patients (18%) received two drug periods. Seven patients (4%) received three drug periods of treatment, ten patients (6%) received four drug periods, and 13 patients (8%) received five or more drug periods (Figure 4). The drugs prescribed in the program included adalimumab, infliximab, ixekizumab, secukinumab, ustekinumab, risankizumab, guselkumab, tildrakizumab, and bimekizumab.

**Figure 5:**
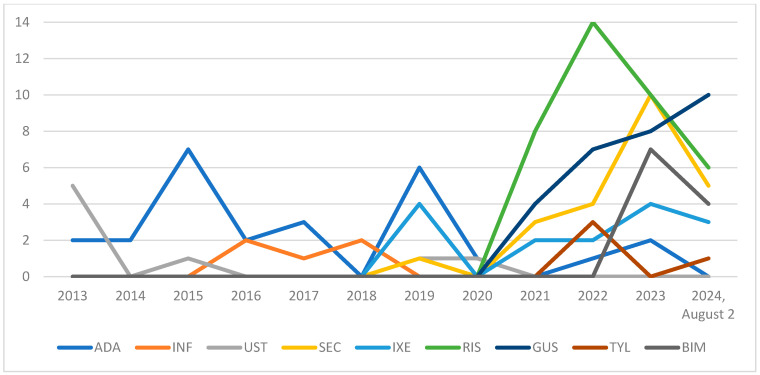
Cumulative recruitment of patients into the B.47 program, stratified by the type of biologic therapy prescribed (*N* = 159). ADA—adalimumab, INF—infliximab, UST—ustekinumab, SEC—secukinumab, IXE—ixekizumab, RIS—risankizumab, GUS—guselkumab, TYL—tildrakizumab, and BIM—bimekizumab.

As shown in Figure 5, recruitment to the program has increased dramatically since 2021, with higher numbers recorded each subsequent year. The most frequently introduced drug in first-line treatment was risankizumab (38 patients), followed by guselkumab (29 patients), adalimumab (26 patients), and secukinumab (23 patients) (Figure 5).

**Figure 6:**
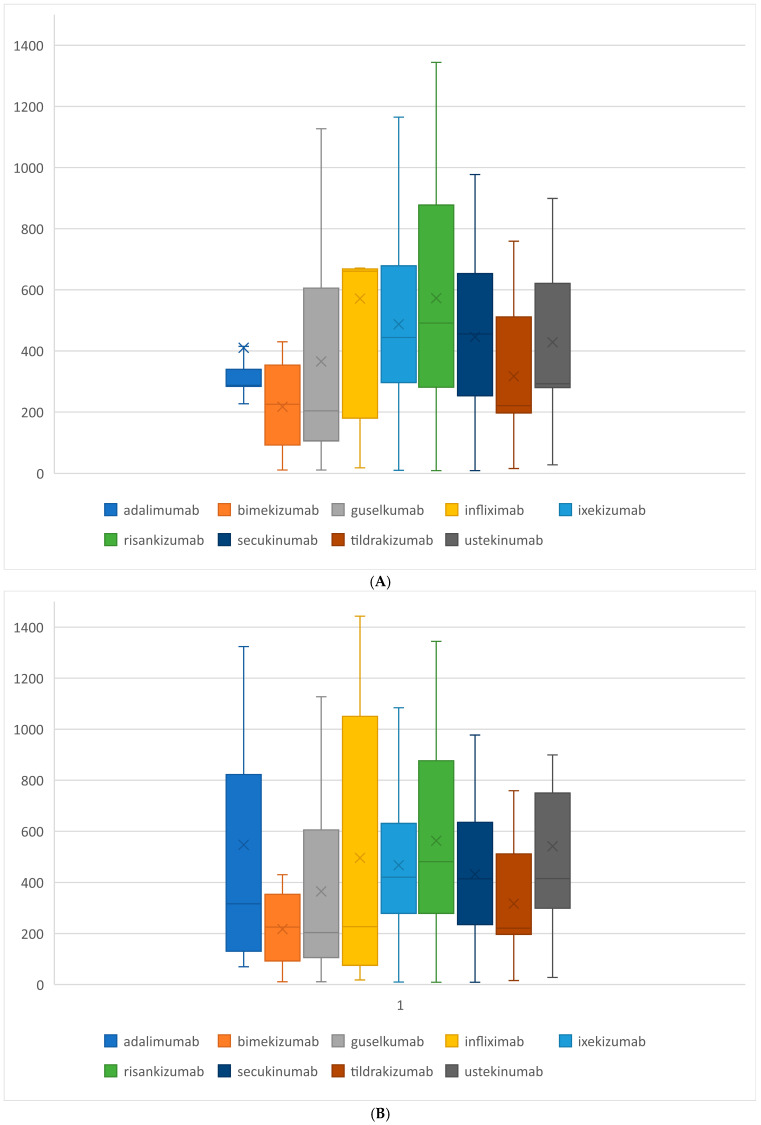
The duration of treatment in the B.47 program, (**A**) including administratively concluded cycles (*N* = 295) and (**B**) excluding administratively concluded cycles (*N* = 216).

Overall, adalimumab and risankizumab were the most commonly used biologics, each with 68 treatment periods. The total exposure time for adalimumab was 27,898 days, and for risankizumab, it was 38,927 days. On average, the duration of a drug period was 443.42 days. Excluding administratively ended cycles, the mean duration increased to 463.14 days. Notably, the biggest differences between the two methods of calculating the mean duration of the drug period were observed for adalimumab and ustekinumab. The mean durations without ended cycles were higher: 410.3 vs. 547 days (*p* = 0.0458) for adalimumab, and 428.3 vs. 541.3 days (*p* = 0.314) for ustekinumab. In contrast, for infliximab, the mean duration was lower when excluding ended cycles: 571.11 vs. 495.8 days (*p* = 0.198). The duration of the drug period for each drug is summarized in Table 4 and Figure 6.

**Figure 7:**
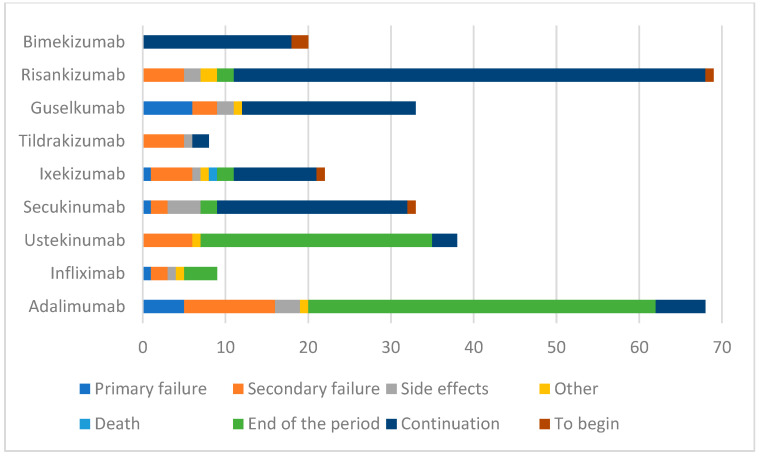
Reasons for treatment discontinuation (*N* = 300).

In the analyzed group, treatment was discontinued due to primary failure in 17 drug periods and secondary failure in 40 drug periods, and discontinuation was the patient’s decision in six cases. Secondary failures were observed most often for tildrakizumab (five out of eight treated patients experienced a loss of adequate response). The lowest rates of secondary failures were observed for bimekizumab (0%), secukinumab (6.06%), risankizumab (7.25%), and guselkumab (9.09%). Side effects led to treatment discontinuation in 14 drug periods (4.67%), as summarized in Table 5 and Figure 7. One patient died during treatment; the cause of death was not related to the biological treatment. The most common reasons for drug discontinuation were eczema in five cases and an allergic reaction at the site of injection in two cases (Table 6).

## 4. Discussion

The majority of patients in our analysis had at least one comorbidity and at least one challenging-to-treat area involved. More than one-third of patients received multiple treatment cycles, with anti-IL-23 agents being the most commonly prescribed. Notably, bimekizumab was the only drug to achieve a 100% treatment success rate.

The Polish Drug Program B.47 is a government-funded therapeutic initiative aimed at providing access to biological treatments for patients with moderate-to-severe psoriasis who have not responded adequately to conventional therapies [4]. The program assumptions, eligibility criteria, course of treatment, and termination criteria are described in the Supplementary Materials. According to the NHS, as of 1 December 2024, 63 sites in Poland are conducting treatment within the program [5]. By 2 August 2024, our site had enrolled 159 patients treated with 300 drug periods. Of these patients, two were children, both treated with one drug period of adalimumab.

Psoriasis is often associated with a range of comorbidities, including psoriatic arthritis and an increased risk of metabolic syndrome, such as obesity, type 2 diabetes mellitus, dyslipidemia, and cardiovascular diseases. Patients with psoriasis are also at a higher risk of developing mental health disorders, non-alcoholic fatty liver disease, Crohn’s disease, and uveitis [1]. Data on the comorbidities analyzed in our group were limited, so we only included obesity, diabetes mellitus, arterial hypertension, and dyslipidemia. Joint pain or oedema was reported by over a third of the patients, but since they did not undergo diagnostic tests, the symptoms could not be diagnosed as psoriatic arthritis. A large proportion of our patients had abnormal body weight, with nearly 38% being overweight and 39% being obese. Obesity is a chronic, low-grade inflammatory condition characterized by the release of pro-inflammatory cytokines from adipose tissue, including TNF-α, IL-6, and IL-17, which can impact treatment outcomes. Evidence from the literature indicates that obesity adversely affects the clinical response to biological therapies in patients with psoriasis, with anti-IL-17 and anti-IL-23 therapies showing greater susceptibility to reduced efficacy in individuals with a higher BMI compared to anti-TNF agents. The mean BMI in our cohort was 28.9, which is similar to that of the German Psoriasis Registry PsoBest cohort and lower than the British Association of Dermatologists Biologics and Immunomodulators Register (BADBIR) cohort (28.7 and 31.0, respectively). The frequency of analyzed comorbidities in our group was higher than in the two previously mentioned populations [6,7].

Some specific areas of the body are usually challenging to manage effectively in psoriasis. These areas include the scalp, nails, genitals, face, palms/soles, and skin folds. The frequency of involvement of these sites varies in the literature, typically ranging as follows: scalp, 40–60%; nails, 20–60%; genital area, 15–40%; face, 20–30%; palmoplantar, 10–20%; and intertriginous, 20–30% [8,9,10,11]. In our population, the vast majority had at least one of these areas affected, most frequently the scalp. As known, these areas are not only more challenging to treat effectively but can also have a significant impact on a patient’s quality of life, even if the overall disease severity is relatively low [12]. Biological drugs, particularly IL-23 and IL-17 inhibitors, have been shown to be effective in challenging areas such as the scalp, nails, anogenital region, and palmoplantar areas, which are often less responsive to conventional therapies [13,14]. The introduction of specific-location eligibility criteria to the B.47 program in 2021 allowed 40 patients to enroll in biological treatment, regardless of their BSA and PASI scores. This change in the program provides a unique opportunity for funded access to advanced therapies for those who suffer from significant discomfort, functional impairment, or psychological distress due to psoriasis in these sensitive or visible areas.

Biological treatment of psoriasis within the B.47 program differs in several key aspects from biological treatment in other countries and has historically been more rigid in terms of treatment duration and drug availability. The eligibility criteria are based not only on the severity of psoriasis but also on the failure of conventional therapies. Guidelines and criteria in other countries might be more stringent or flexible, leading to differences in access to biological treatments. In our group, the average duration of psoriasis before starting biological treatment in the B.47 program was 18.2 ± 12.85 years, which is similar to the Italian cohort (18.6 ± 13.3) [15]. This is shorter compared to the UK cohort of psoriasis patients receiving biological therapies registered in the BADBIR registry, where the average duration was 21.7 ± 12.6 years (*n* = 9201), and also shorter compared to the German cohort of psoriasis patients receiving biological therapies registered in the PsoBest registry, where the average duration was 21.9 ± 14.1 years [6,7].

In the analyzed group, the effectiveness of standard systemic methods (methotrexate, ciclosporin A, and PUVA) was quite poor, and the use of these drugs resulted in side effects. The occurrence and profile of side effects are in agreement with the Summary of Product Characteristics [16,17,18]. Methotrexate cessation was most often caused by nausea and vomiting, diarrhea, abdominal pain, hepatotoxicity, general fatigue, and a loss of appetite leading to weight loss (approx. 61% of methotrexate users). For ciclosporin A, arterial hypertension, gastrointestinal issues, or headaches and dizziness lead to treatment termination in about two-thirds of users. About the same proportion of patients receiving acitretin stopped treatment because of excessive skin and mucosa dryness, hair loss, lipid abnormalities, and psoriasis flare-ups. It should be considered that the effectiveness and side effects of these treatments differ from those observed in the general population, as this is a specific group of patients enrolled to receive biological treatments after experiencing ineffectiveness, intolerance, or contraindications to standard therapies.

On the contrary, treatment with biologics was generally well tolerated, with side effects leading to treatment discontinuation in only 14 cases. For three patients, eczematous lesions were the reason for discontinuation—specifically, with adalimumab, secukinumab, and risankizumab. According to recent data, the overall incidence of paradoxical eczema following the use of biological drugs for psoriasis is low, with TNF inhibitors most frequently causing paradoxical eczema [19]. Severe injection site reactions led to discontinuation in one patient treated with adalimumab and one with guselkumab. According to the literature, among the anti-psoriatic biologics available in the B.47 program, the highest prevalence of injection site reactions was reported with etanercept (11.4%) and ixekizumab (11.2%). Conversely, the biologics with the lowest prevalence of injection site reactions were risankizumab (0.8%), guselkumab (1.3%), and secukinumab (1.9%) [20]. In our database, only severe injection site reactions were reported, and given the relatively small size of our group, these results may not be generalizable.

Another difference between the B.47 program and biological treatment in other countries lies in drug availability, as access to various biologics can vary between countries due to differences in regulatory approvals and reimbursement policies. Consequently, the maximum observation period for bimekizumab is the shortest (430 days), making it challenging to compare its drug survival with that of biologics that have been available for a longer time. Additionally, until changes were implemented in 2023, the B.47 program had predefined termination periods for each drug, whereas in many other countries, biological treatments for psoriasis are often continued without set time limits. Therefore, the authors decided to calculate the duration of treatment for each active substance in two ways: once including all drug periods, and once excluding the periods that were administratively terminated. Given that in the early years of the program, the treatment periods for adalimumab and ustekinumab were set to end at the 48-week mark, it is not surprising that the mean duration for these drugs differed significantly between the two methods of calculation.

Moreover, we observed an increase in enrollment from 2021, which may be explained by changes in the program’s enrollment criteria, including the introduction of specific-location eligibility, as well as lowering of the DLQI, BSA, and PASI thresholds. Additionally, the COVID-19 pandemic may have played a role. It is known that infections can exacerbate psoriasis, and vaccinations should also be considered. For example, COVID-19 vaccinations can induce a hyperinflammatory state, which may contribute to psoriasis flares [21].

## 5. Conclusions

The patient population enrolled in the B.47 program reflects a typical psoriasis demographic, with a slightly higher prevalence of males and an average disease duration of over 18 years before starting biological treatment. A significant portion of patients had comorbid conditions, highlighting the importance of managing not only psoriasis but also associated metabolic and cardiovascular risks. Scalp and nail involvement were common in the cohort, indicating the need for treatments that are effective for specific, difficult-to-treat areas.

The low success rate and frequency of side effects illustrate the limitations of traditional therapies and the critical role biologics play in providing more effective long-term control. The variability in treatment durations and secondary failure rates across different biologics suggests that, although biologics offer improved outcomes, personalized treatment choices are important for sustained effectiveness. The analysis of treatment duration, both including and excluding administratively terminated cycles, highlights the impact of structured treatment programs. The recent shift to potentially life-long biological treatment represents an important advancement, allowing for sustained disease management.

This research provides an important perspective on the topic, but several limitations need to be acknowledged when interpreting the findings. First, as a single-center study, the results may not be fully representative of the broader population of patients with plaque psoriasis in Poland or internationally. Second, the relatively small sample size limits the generalizability of the results and may reduce the statistical power to detect subtle differences in treatment patterns or outcomes. Another limitation is the retrospective nature of the study, which may be subject to incomplete or inaccurate data collection. Moreover, the analysis of treatment duration was complicated by the administrative termination periods present in the B.47 program before 2023, which may have impacted the observed drug survival times. Future cross-sectional studies with larger cohorts and prospective designs would be valuable in validating these findings and expanding knowledge about biological treatment in patients with plaque psoriasis in Poland.

This study, by examining data from a real-world cohort, sought to highlight the unique aspects of psoriasis management in the Polish population. Understanding patient profiles and treatment patterns is essential for optimizing care, aligning treatment approaches with international standards, and addressing specific needs and challenges within the Polish healthcare context. Further research is set to analyze treatment patterns and outcomes in more detail, with a focus on understanding how different therapies are utilized across diverse patient populations. In addition, future studies will aim to identify key prognostic factors that influence treatment efficacy, providing deeper insights into optimizing psoriasis treatment strategies.

## Figures and Tables

**Table 1:** Patient demographics and baseline characteristics (*N* = 146).

Variable	Value
Total number of patients, *n*	146
Sex, female, *n* (%)	65 (44.52)
Ethnicity, white, *n* (100%)	146 (100)
Age of onset (years, mean ± SD)	25.6 ± 13.45
Age at biological treatment commencement (years, mean ± SD)	48 ± 14.70
Disease duration prior to biological treatment (years, mean ± SD)	18.2 ± 12.85
Family history of psoriasis, *n* (%)	66 (45.21)
BMI (kg/m^2^, mean ± SD)	28.9 ± 5.86
Underweight (BMI < 18.5)	3 (2.05)
Normal (BMI 18.5–24.9)	31 (21.23)
Overweight, *n* (%)	55 (37.67)
Obesity, *n* (%)	57 (39)
Obesity Class I (BMI: 30.0–34.99), *n* (%)	37 (25.34)
Obesity Class II (BMI: 35.0–39.99), *n* (%)	12 (8.22)
Obesity Class III (BMI: ≥ 40), n (%)	8 (5.48)
Dyslipidemia, *n* (%)	40 (24.40)
Arterial hypertension, *n* (%)	54 (37)
Current or former smoking, *n* (%)	27 (18.49)
Type 2 diabetes mellitus, *n* (%)	23 (15.75)
Joint pain, *n* (%)	50 (34.25)
One comorbidity, *n* (%)	40 (27.40)
Two comorbidities, *n* (%)	25 (17.12)
Three comorbidities, *n* (%)	12 (8.22)
Four comorbidities, *n* (%)	12 (8.22)

**Table 2:** Baseline severity and pattern of psoriasis (*N* = 146).

Baseline DLQI score	19.38 ± 6.06
Baseline BSA score	23.08 ± 15.20
Baseline PASI score	17.39 ± 8.24
Scalp, *n* (%)	109 (74.66)
Nails, *n* (%)	94 (64.38)
Genital area, *n* (%)	44 (30.14)
Face, *n* (%)	29 (19.86)
Palms and soles, *n* (%)	27 (18.49)
Intertriginous areas, *n* (%)	20 (13.70)
Specific areas enrollment, *n* (%)	40 (27.40)

**Table 3:** History of previous non-biological systemic treatment modalities (*N* = 146).

Methotrexate	
Number of patients, *n* (%)	126 (86.30)
Effectiveness, *n* (%)	13 (10.31)
Side effects, *n* (%)	70 (55.56)
Ciclosporin A	
Number of patients, *n* (%)	114 (78.08)
Effectiveness, *n* (%)	15 (13.16)
Side effects, *n* (%)	73 (64.04))
Acitretin	
Number of patients, *n* (%)	48 (32.88)
Effectiveness, *n* (%)	1 (2.08)
Side effects, *n* (%)	28 (58.33)
PUVA	
Number of patients, *n* (%)	33 (22.76)
Effectiveness, *n* (%)	3 (9.09)
Side effects, *n* (%)	11 (33.33)
UVB-NB	
Number of patients, *n* (%)	58 (39.73)
Effectiveness, *n* (%)	20 (34.48)
Side effects, *n* (%)	3 (5.17)

**Table 4:** Duration of the treatment in the B.47 program in days. Column 1: including administratively concluded cycles (*N* = 295). Column 2: without administratively concluded cycles (*N* = 216).

Exposition	Drug Periods		Mean		Min		Max		SD		Total Exposure Time	
	1	2	1	2	1	2	1	2	1	2	1	2
Total	295	215	443.42	463.14	9	9	1977	1977	333.08	371.31	131,251	99,576
Adalimumab	68	26	410.26	547.08	70	70	1977	1977	360.78	543.80	27,898	14,221
Infliximab	9	5	571.11	495.81	18	18	1443	1443	421.24	582.17	5140	2479
Ustekinumab	38	10	428.26	541.30	28	28	1428	1428	253.69	393.00	16,274	5413
Secukinumab	32	30	445.88	431.27	9	9	977	977	269.61	272.33	14,268	12,938
Ixekizumab	21	19	487.33	467.21	10	10	1165	1165	324.23	335.14	10,234	8877
Tildrakizumab	8	8	317.25	317.25	16	16	759	759	237.46	237.46	2538	2538
Guselkumab	33	33	365.39	365.39	11	11	1127	1127	339.59	339.59	12,058	12,058
Risankizumab	68	66	572.46	562.70	9	9	1344	1344	357.88	356.58	38,927	37,138
Bimekizumab	18	18	217.44	217.44	11	11	430	430	140.87	140.87	3914	3914

**Table 5:** Reasons for treatment discontinuation *n* (%), (*N* = 300).

	Primary Failure	Secondary Failure	Side Effects	Other	Death	End of the Period	Continuation	To Begin	Total
Adalimumab	5 (7.35)	11 (16.18)	3 (4.41)	1 (1.47)	0	42 (61.76)	6 (8.82)	0	68 (22.67)
Infliximab	1 (11.11)	2 (22.22)	1 (11.11)	1 (11.11)	0	4 (44.44)	0	0	9 (3.00)
Ustekinumab	0	6 (15.79)	0	1 (2.63)	0	28 (73.68)	3 (7.89)	0	38 (12.67)
Secukinumab	1 (3.03)	2 (6.06)	4 (12.12)	0	0	2 (6.06.)	23 (69.70)	1 (3.03)	33 (11.00)
Ixekizumab	1 (4.55)	5 (22.73)	1 (4.55)	1 (4.55)	1 (4.55)	2 (9.09)	10 (45.45)	1 (4.55)	22 (7.33)
Tildrakizumab	0	5 (62.50)	1 (12.50)	0	0	0	2 (25.00)	0	8 (2.67)
Guselkumab	6 (18.18)	3 (9.09)	2 (6.06)	1 (3.03)	0	0	21 (63.64)	0	33 (11.00)
Risankizumab	0	5 (7.25)	2 (2.90)	2 (2.90)	0	2 (2.90)	57 (82.61)	1 (1.45)	69 (23.00)
Bimekizumab	0	0	0	0	0	0	18 (90.00)	2 (10.00)	20 (6.67)
Total	14 (4.67)	39 (13.00)	14 (4.67)	7 (2.33)	1 (0.33)	80 (26.67)	140 (46.67)	5 (1.67)	300

**Table 6:** Side effects of the biological drugs (number of patients in brackets).

Adalimumab (3)
Swelling, erythema, itch at the injection site (1)
Eczema, soles (1)
Pseudoscleroderma (1)
Infliximab (1)
Hepatotoxicity (1)
Secukinumab (4)
Eczema, palms (2)
Recurrent bacterial and fungal infections, erythema nodosum (1)
Headache after injection (1)
Ixekizumab (2)
Nasuea, vomiting after injection (1)
Tildrakizumab (1)
Eczema, diffuse (1)
Guselkumab (2)
Swelling, erythema, itch at the injection site (1)
Stomach pain (1)
Risankizumab (2)
Eczema, palms (1)
Adnexitis, eosinophilic enteritis (1)

## Data Availability

The data presented in this study are available on request from the corresponding author.

## Supplementary Materials

The following supporting information can be downloaded at: https://www.mdpi.com/article/10.3390/jcm13247647/s1, Figure S1. Changes in B.47 program; Table S1. Side effects of methotrexate (*N* = 126); Table S2. Side effects of ciclosporin A (*N* = 114); Table S3. Side effects of acitretin (*N* = 48).
