# Supplementary material for: Demographics, Disease Characteristics, and Treatment Patterns of Patients with Plaque Psoriasis Treated with Biological Drugs: The Experience of a Single-Centre Study in Poland
Source: J Clin Med. 2024 Dec 16;13(24):7647. doi: 10.3390/jcm13247647 (PMC11727830; doi:10.3390/jcm13247647)
Supplement: Supplementary file 1 [file jcm-13-07647-s001.zip › jcm-3327797-supplementary.pdf]

Journal of Clinical Medicine

Supplementary material A

B.47 program summary

The Polish Drug Program B.47 “Treatment of moderate to severe plaque psoriasis (ICD-10 L40.0)” aims to provide access to biologic therapies for patients with moderate to severe plaque psoriasis who have demonstrated an inadequate response, intolerance, or contraindications to at least two standard systemic therapies, such as phototherapy, methotrexate, cyclosporine, or acitretin. The eligibility criteria for the B.47 drug program for psoriasis have changed over time.

To be eligible for enrollment in the B.47 program, patients must have an inadequate response, intolerance, or contraindications to at least two standard systemic therapies. These therapies include: acitretin at a dose of no less than 0.5 mg/kg body weight/day, evaluated after two months; methotrexate at a dose of at least 15 mg/week, evaluated after three months; ciclosporin A at a dose of 3 to 5 mg/kg body weight/day, evaluated after three months; and PUVA (psoralen + UVA), also evaluated after three months.

Starting in 2013, the eligibility criteria for all drugs required a Daily Life Quality Index (DLQI) and body surface area (BSA) score of at least 10, and a Psoriasis Area and Severity Index (PASI) score of at least 18. From 2015 to 2021, the criteria included a DLQI and BSA score of at least 10, and a PASI score of at least 10 for infliximab, while a PASI score of at least 18 was required for other available drugs. Between 2021 and 2023, the program required a DLQI and BSA score of at least 10, with a PASI score of at least 10 for TNF-alpha inhibitors or tildrakizumab, and a PASI score of at least 18 for IL-17 inhibitors, ustekinumab, guselkumab, or risankizumab. Currently, from 2023 onwards, patients must have a confirmed diagnosis of moderate to severe plaque psoriasis, with a DLQI, BSA and PASI score of at least 10 to qualify for all the substances available in the program. In addition, since 2021, the patients might be also qualified for biological treatment if psoriatic lesions affect specific areas, such as the scalp, face, genital area, palms and or nails, regardless BSA and PASI score. Finally, children aged 6 and older have been eligible for the program since 2014, and the eligibility was extended to children aged 4-5 years in 2023.

Medications currently available under the program include TNF-inhibitors (adalimumab, etanercept, infliximab, certolizumab pegol), IL-17 inhibitors (ixekizumab, secukinumab), IL-12/23 inhibitor - ustekinumab, IL-23 inhibitors (risankizumab, guselkumab, tildrakizumab), and IL-17AF inhibitor - bimekizumab. Up until 2018, only ustekinumab and TNF-alpha inhibitors (adalimumab, etanercept, and from 2015 infliximab) were available in the program. From 2018, ixekizumab and secukinumab became available. In 2020, guselkumab and risankizumab were added. In 2021, certolizumab pegol and tildrakizumab became available. The most recent addition to the program is bimekizumab, which has been available since March 1, 2023.

Patients enrolled in the B.47 program are subject to ongoing monitoring to assess the effectiveness and safety of the treatment. This includes performing following blood tests: complete blood count (CBC), C-reactive protein (CRP) level, serum creatinine level, aspartate aminotransferase (AST) and alanine aminotransferase (ALT) levels. The evaluations occur after two months (± 30 days) and four months (± 30 days) after the first administration of the active substance. Additionally, four months (± 30 days) after the first administration of the active substance, an assessment of the effectiveness of the therapy should be conducted using the PASI, DLQI, and BSA indices. Adequate Response to Treatment is defined as: a reduction in the PASI score by at least 75% or a reduction in the PASI score by at least 50%, along with an improvement in quality of life as assessed by the DLQI scale by at least 5 points. If the therapy is continued, the above laboratory tests and clinical effectiveness assessment should be repeated at least once every six months (± 30 days).

Changing the therapy to a different active substance listed in the program is only possible in the following situations: the occurrence of a severe allergic reaction to the substance, or a presence of adverse effects that prevent the continuation of therapy or a failure to achieve an adequate response to the administered active substance after four months (± 30 days) – primary failure, or loss of adequate response observed during two consecutive visits – secondary failure. The drug program's description has evolved over the years to enhance patient access to effective treatment. Until 2018, treatment durations varied by drug: infliximab, secukinumab, and ixekizumab were administratively terminated after 96 weeks; ustekinumab and adalimumab after 48 weeks; and etanercept after 24 weeks. Between 2018 and 2023, the treatment period was standardized to 96 weeks for all drugs. Starting in 2023, treatment can now be continued indefinitely, as long as effective and safe.

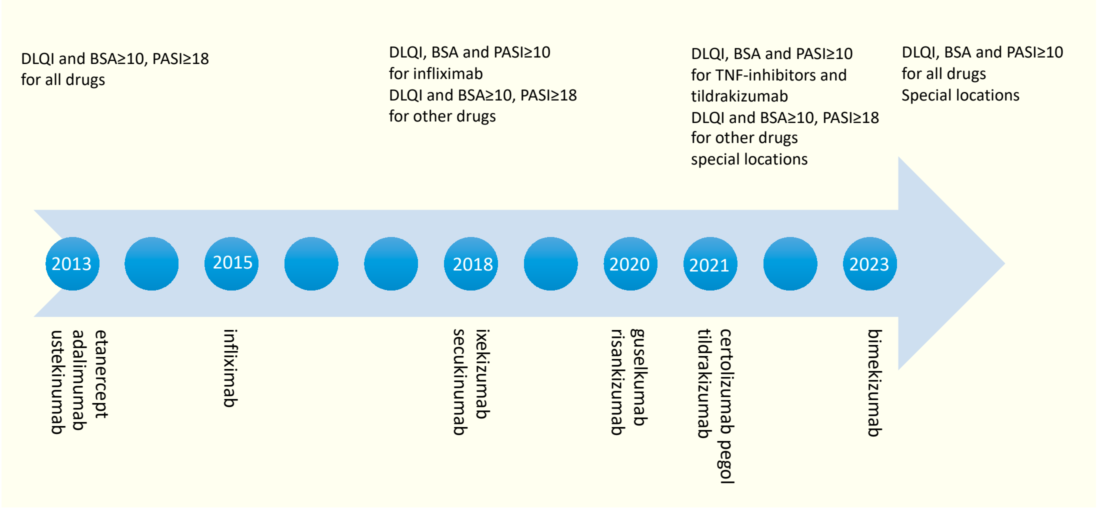

Figure S1. Changes in B.47 program.
Ref. NHS drug programs [Internet]. [cited 2024 Dec 1]. Available from: https://www.gov.pl/web/zdrowie/programy-lekowe

Supplementary material B
Side effects of standard treatment methods

Table S1. Side effects of methotrexate (N=126).

**Variable**	**Value, n (%)**
Gastrointestinal issues (nausea, vomiting, diarrhea, abdominal pain)	35 (27.78)
Elevated liver enzymes	24 (19.05)
General fatigue, loss of appetite, weight loss	18 (14.29)
Headache, dizziness	11 (8.73)
Hair loss	8 (6.35)
Metallic taste in mouth	2 (1.59)
Depression	2 (1.59)
Dyspnoea	2 (1.59)
Gastrointestinal bleeding	2 (1.59)
Decreased libido	1 (0.79)
Ulcerations in the mouth	1 (0.79)
Chest pain	1 (0.79)
Upper respiratory tract infections	1 (0.79)
Hypertension	1 (0.79)
Kidney impairment	1 (0.79)
Menstrual disorders	1 (0.79)
Visual disturbances	1 (0.79)
Body stiffness	1 (0.79)
Pulmonary fibrosis	1 (0.79)
Mycosis fungoides	1 (0.79)
Chronic cough	1 (0.79)
Recurrent herpetic infections	1 (0.79)

Leukopenia, neutropenia	1 (0.79)

Table S2. Side effects of ciclosporin A (N=114).

**Variable**	**Value, n (%)**
Hypertension	45 (39.47)
Gastrointestinal issues (nausea, vomiting, diarrhea, abdominal pain)	15 (13.16)
Headache, dizziness	15 (13.16
Fatigue, loss of appetite	9 (7.89)
Paresthesia	6 (5.26)
Hand tremors	6 (5.26)
Depression, irritability, anxiety	5 (4.39)
Kidney impairment	5 (4.39)
Gum hyperplasia and bleeding	4 (3.51)
Hypertrichosis	4 (3.51)
Muscle pain or cramps	3 (2.63)
Dyslipidemia	2 (1.75)
Upper respiratory tract infections	2 (1.75)
Elevated liver enzymes	2 (1.75)
Leg swelling	2 (1.75)
Palpitations, pain in the chest	2 (1.75)
Gastrointestinal bleeding	2 (1.75)
Sleeping disorders	1 (0.88)
Hot flashes	1 (0.88)
Haemoptysis	1 (0.88)
Anaemia	1 (0.88)
Urinary retention	1 (0.88)
Hair loss	1 (0.88)
Lymphadenitis	1 (0.88)
Hyperhidrosis	1 (0.88)

Table S3. Side effects of acitretin (N=48).

**Variable**	**Value, n (%)**
Skin and mucosa dryness	12 (25)
Hair loss	7 (14.58)
Dyslipidemia	6 (12.5)
Exacerbation of psoriasis	5 (10.42)
Gastrointestinal issues (nausea, vomiting, diarrhea, abdominal pain)	3 (6.25)
Headache, dizziness	2 (4.17)
Fatigue, loss of appetite	2 (4.17)
Hands erosions	1 (2.08)

Epistaxis	1 (2.08)
Paronychia	1 (2.08)
Hypersensitivity to the sun	1 (2.08)
Chronic cough	1 (2.08)
Visual disturbances	1 (2.08)
Eczema	1 (2.08)
Periungual granulomas	1 (2.08)
